# Induction of apoptosis through oxidative stress-related pathways in MCF-7, human breast cancer cells, by ethyl acetate extract of *Dillenia suffruticosa*

**DOI:** 10.1186/1472-6882-14-55

**Published:** 2014-02-14

**Authors:** Yin Sim Tor, Latifah Saiful Yazan, Jhi Biau Foo, Nurdin Armania, Yoke Kqueen Cheah, Rasedee Abdullah, Mustapha Umar Imam, Norsharina Ismail, Maznah Ismail

**Affiliations:** 1 Laboratory of Molecular Biomedicine, Institute of Bioscience, Universiti Putra Malaysia, 43400 UPM, Serdang, Selangor, Malaysia; 2 Department of Biomedical Science, Faculty of Medicine and Health Sciences, Universiti Putra, Malaysia, 43400 UPM, Serdang, Selangor, Malaysia; 3 Department of Veterinary Pathology and Microbiology, Faculty of Veterinary Medicine, Universiti, Putra Malaysia, 43400 UPM, Serdang, Selangor, Malaysia

**Keywords:** *Dillenia suffruticosa*, Breast cancer, Cytotoxic, Apoptosis, Oxidative stress pathway

## Abstract

**Background:**

Breast cancer is one of the most dreading types of cancer among women. Herbal medicine has becoming a potential source of treatment for breast cancer. Herbal plant *Dillenia suffruticosa* (Griff) Martelli under the family Dilleniaceae has been traditionally used to treat cancerous growth. In this study, the anticancer effect of ethyl acetate extract of *D. suffruticosa* (EADs) was examined on human breast adenocarcinoma cell line MCF-7 and the molecular pathway involved was elucidated.

**Methods:**

EADs was obtained from the root of *D. suffruticosa* by using sequential solvent extraction. Cytotoxicity was determined by using MTT assay, mode of cell death by cell cycle analysis and apoptosis induction by Annexin-FITC/PI assay. Morphology changes in cells were observed under inverted light microscope. Involvement of selected genes in the oxidative stress-mediated signaling pathway was explored using multiplex gene expression analysis.

**Results:**

The treatment of EADs caused cytotoxicity to MCF-7 cells in a dose- and time-dependent manner at 24, 48 and 72 hours with IC_50_ of 76 ± 2.3, 58 ± 0.7 and 39 ± 3.6 μg/mL, respectively. The IC_50_ of tamoxifen-treated MCF-7 cells was 8 ± 0.5 μg/mL. Induction of apoptosis by EADs was dose- and time- dependent. EADs induced non-phase specific cell cycle arrest at different concentration and time point. The multiplex mRNA expression study indicated that EADs-induced apoptosis was accompanied by upregulation of the expression of *SOD1*, *SOD2*, *NF*-*κB*, *p53*, *p38 MAPK*, and *catalase*, but downregulation of *Akt1*.

**Conclusion:**

It is suggested that EADs induced apoptosis in MCF-7 cells by modulating numerous genes which are involved in oxidative stress pathway. Therefore, EADs has the potential to act as an effective intervention against breast cancer cells.

## Background

Breast cancer is the most typical cancer diagnosed among women thus far [1]. The mortality rate of breast cancer declines over the year and the latest five years data affirm that breast cancer incidence rate is stable [2]. Death rate in female patients reduces by 15.3% since year 1991 due to amelioration in early detection and treatment [3]. Nevertheless, breast cancer cases are still accounting for 23% of total new cancer cases globally [4]. With advance in molecular knowledge, more novel anti-cancer agents with great selectivity and specificity ought to be developed to overcome limitation of current treatments.

Deregulated cell proliferation and inhibition of cell death evoke uncontrolled development of cancer [5]. Cancer cells exhibit resistance to apoptosis or cell death in order to survive and metastasize [6]. The common feature of cancer progression, inclusive of breast cancer, is genetic alteration in apoptotic pathways such as alteration of pro- and anti-apoptotic genes that provides an insight to a target of treatment [7]. The treatment strategy aims to destroy cancer cells by activating their apoptotic signaling pathways, ideally to induce selective apoptosis cell death on cancer cells, and exert no harmful effects on normal cells [8,9].

Apoptosis form of cell death is chosen to eliminate cancer cells instead of other alternative mechanism because it is a series of regulated cell events that perform cellular suicide without triggering inflammatory response, and neither harmful to neighboring cells. It differs from necrosis that causes membrane rupture thus elicits inflammatory response [10]. Most importantly, apoptotic form of cell death is occasionally altered in cancer cells. The understanding of apoptosis unfolds a gate to tumor-specific apoptosis therapy [11].

Reactive oxygen species (ROS) such as hydroxyl radical (OH^-^), superoxide anions (O_2_^-^), hydrogen peroxide (H_2_O_2_) and peroxyl radicals (ROO^-^) are common products of aerobic metabolism that can be useful or harmful to biological system [12]. Low concentration of ROS may facilitate signal transduction, enzyme activation and other cellular functions, but high concentration of ROS generates damage to DNA, protein and lipid which can lead to cells transformation such as cancer [13,14]. To offset the ROS detrimental effect, cells are complement with antioxidant defense system transformation that includes superoxide dismutases, catalase, glutathione and others as protective mechanism [15]. In fact, ROS and antioxidants exist in balance under normal circumstances. When the equilibrium between ROS and anti-oxidants is disrupted, collective generation of ROS is described as oxidative stress [16]. Evidence showed that ROS not only function as regulator of subcellular events but are also able to induce cell death through apoptotic pathway [17]. Recently, many anticancer agents such as 5-fluorouracil, tamoxifen and paclitaxel exploit this channel to eliminate cancer cells by continually exert cellular ROS to a threshold that can kill cancerous cells effectively [14,18-20].

Plant has long history as a source of anticancer agents, and gives prominent impact on modern drug development process [21]. Over the last 20 years, exceeding 25% of drugs are plant derived while another 25% are originated from chemically modified natural products [22]. Plant-based anticancer drugs such as etoposide (topoisomerase II inhibitor) from epipodophyllotoxins, topotecan and irinotecan (topoisomerase I inhibitor) from camptothecins, vincristine and vinblastine (tubulin-binding agent) from vinca alkaloids, induce apoptosis in chemotherapeutic therapy against various types of cancer [23,24]. The main target of action for those anti-cancer drugs may be distinct, but eventually they lead to identical cell death pathway, which is apoptosis [25,26]. Therefore, in order to discover more plant-based anticancer agent, various plant extracts were investigated for their apoptosis-inducing ability. Interestingly, although plants are frequently reported to possess antioxidant activity, some of them are found to exert distinguish apoptosis inducing ability through the induction of oxidative stress [27-30].

*Dillenia suffruticosa* (Griff) Martelli (*D. suffruticosa*), which belongs to family Dilleniaceae, is a plant native in Peninsular Malaysia, Kalimantan, Sumatra and Singapore [31,32]. The evergreen shrub can be found in secondary forest and swampy ground. The fruit of the plant has the ability to treat cancerous growth [33]. Other traditional use of the plant is to relieve rheumatism [34]. Methanolic extract of *D. suffruticosa* showed a broad spectrum of antimicrobial activity against *Bacillus cereus*, *Bacillius subtilis*, *Candida albicans*, and *Pseudomonas aeruginosa*[35]. Water extract of *D. suffruticosa* also exhibited inhibitory action against replication of dengue virus type 2 [36]. Armania *et al*. [37] reported that extract of *D. suffruticosa* showed high antioxidant and cytotoxic activities towards various cell lines including Hela, MCF-7, MDA-MB-231, A549 and HT-29 cell lines. In this study, root extract was selected for elaborated study. As the previous study demonstrated that root extract of the plant exhibited the most potent cytotoxic activity, in comparison to fruit, leaf, and flower parts of the plant.

The aim of this study was to investigate the anticancer effect of ethyl acetate of *D. suffruticosa* (EADs) in breast cancer cells, MCF-7, and to explore the apoptotic signaling pathway underlying it.

## Methods

### Chemicals and reagents

Hexane, dichloromethane, ethyl acetate and dimethyl sulfoxide (DMSO) were purchased from FS Chemicals (Francfort, Germany) (analytical grade). RPMI 1640 was purchased from Nacalai Tesque (Kyoto, Japan). Fetal bovine serum, trypsin, streptomycin and penicillin were obtained from PAA Laboratories GmBH (Pasching, Austria). 3-(4,5-dimethylthiazol-2-yl)-2,5 diphenyltetrazolium bromide (MTT), propidium iodide and RNAse A were purchased from Sigma (St. Loius, USA). Tissue culture flasks, 6-well plates and 96-well plates were obtained from TPP (Trasadingan, Switzerland). Annexin-V FITC Kit was obtained from eBioscience Inc. (San Diego, USA). Real Genomics Total RNA extraction kit (RBC Biosciences, Taiwan) and GenomeLab GeXP Start Kit (Beckman Coulter, USA) were also procured.

### Cell culture

The human adenocarcinoma breast cancer cell line, MCF-7, and mouse fibroblast cell line, 3T3 were obtained from the American Type and Culture Collection (Rockville, USA). Cells were cultured in RPMI 1640 supplemented with 10% fetal bovine serum and 1% penicillin and streptomycin, and maintained in humidified incubator at 37°C in atmosphere of 5% CO_2_.

### Preparation of EADs

The root powder of *D. suffruticosa* was supplied by Primer Herber Sdn. Bhd. (Malaysia). The plant with voucher specimen number SK1937/11 was deposited in the herbarium of Institute of Bioscience, Universiti Putra Malaysia. Briefly, 100 g of the powder was soaked in 300 mL of hexane at a ratio of 1:3 (w/v) with occasional shaking using a rotary shaker for three times at 3:1:1 day interval. The mixture solvent was collected and filtered using Whatman No. 1 filter paper. The residue was dried in an oven at 40°C and subsequently used for successive extraction of dichloromethane followed by ethyl acetate using the same methods. Lastly, filtered ethyl acetate extract was evaporated using a vacuum rotary evaporator (Buchi, Switzerland) [37]. The yield was weighed and kept at -20°C until required. For subsequent experiment, the stock of EADs in DMSO (30 mg/mL) was used. The final concentration of DMSO was 0.33% in all the extracts prepared. DMSO at 0.33% is non-toxic to the cell line mentioned above [38].

### Cytotoxicity of EADs

Cytotoxicity of EADs on MCF-7 cells was determined by the MTT (3-(4,5-dimethylthiazol-2-Yl)-2,5-diphenyltetrazolium bromide) assay [39]. Briefly, 1x10^5^ of cells were seeded in each well of a 96-well plate. After 24 hours incubation, cells were treated with EADs (3.13 to 100 μg/mL). Untreated control cells were also included. After incubation with EADs for 24, 48 and 72 hours, 20 μL of 5 mg/mL of MTT was added into each well and incubated for 3 hours. Active mitochondria in live cells reduced MTT to crystalline purple blue formazan. The number of living cells was proportionate to the amount of crystalline purple blue formazan produced. After incubation, media in each well was discarded and 100 μL of DMSO was added to solubilize the purple blue formazan. The absorbance was measured with an ELISA plate reader (Biotek, USA) at wavelength of 570 nm, and 630 nm as background. A graph of percentage of cell viability versus concentration of EADs was plotted and the IC_50_ (concentration that inhibits 50% of cell growth compared to control) was determined.

### Cell morphology study of apoptosis by inverted light microscope

Briefly, 3 × 10^5^ of MCF-7 cells were seeded in each well of a 6-well plate. After 24 hours of incubation, cells were treated with EADs at concentration of 25 and 50 μg/mL. Control untreated cells were also included. Morphological changes of cells untreated and treated with EADs were examined under an inverted light microscope (Olympus, Tokyo, Japan) after 24, 48 and 72 hours. The cells were captured at the same spot at different time interval.

### Cell cycle analysis

Briefly, 3 × 10^5^ of MCF-7 cells were seeded in each well of a 6-well plate and treated with EADs at 25 and 50 μg/mL. Control untreated cells were also included. After incubation for 24, 48 and 72 hours, cells were trypsinized and washed with PBS. After centrifugation, cell suspension was resuspended repeatedly into single cells prior fixation with 70% ethanol. Fixed cells were kept at -20°C for at least 2 hours. Later, fixed cells were washed with PBS twice and the supernatant was discarded. Cell pellets were resuspended with 425 μL of PBS in a round bottom tube. Next, 50 μL of RNAse and 25 μL of propidium iodide were added into the cell suspension and incubated for 15 minutes on ice in the dark. FACS Calibur (BD Biosciences, USA) and Cell Quest Pro software (BD Biosciences, USA) was used to determine the cell cycle distribution. A total of 10,000 of cells were acquired each time using FACS Calibur flowcytometer. Flowcytometric data were analyzed using Modfit software and displayed in histogram cell count (у-axis) against DNA content (*x*-axis).

### Annexin V/PI apoptosis assay

Briefly, 3 × 10^5^ of MCF-7 cells were seeded in each well of a 6-well plate and treated with EADs at 25 and 50 μg/mL. Control untreated cells were also included. After incubation for 24, 48 and 72 hours, cells were trypsinized, washed twice with PBS and the supernatant was discarded. The cell pellets were mixed with 185 μL of 1X binding buffer. Next, 5 μL of Annexin-V FITC and 10 μL of propidium iodide (PI) were added into the suspension and incubated at room temperature for 10 minutes in the dark. Subsequently, 300 μL of 1X binding buffer was added prior to measurement using FACS calibur flowcytometer and Cell Quest Pro software (BD Biosciences, USA). Samples were kept on ice. This assay was carried out following manufacturer’s kit from BenderMedsystem (Vienna, Austria). The fluorescence colour was detected through 530 and 585 nm band pass filter. A total of 10,000 cells were acquired. Flowcytometric data were analyzed using FlowJo 7.6 software and displayed in dot plot of Annexin V/FITC (*y*-axis) against PI (*x*-axis).

### Multiplex mRNA expression analysis using GeXP analysis system

#### RNA isolation

Briefly, 3 × 10^5^ of MCF-7 cells were seeded in each well of a 6-well plate and treated with EADs at 25 and 50 μg/mL. Untreated control cells were also included. Untreated and EADs treated cells were trypsinized and washed twice with PBS. RNA extraction was performed using the Real Genomics Total RNA extraction kit (RBC Biosciences, Taiwan).

### Reverse transcription and polymerase chain reaction

Samples were prepared according to the GenomeLab GeXP Start Kit (Beckman Coulter, USA). Briefly, 2 μL of customized reverse primers of the desired genes were mixed with 11 μL of RNA free water, 4 μL of reverse transcription buffer, 1 μL of reverse transcriptase and 1 μL of 50 ng/μL of sample. The reverse transcription reaction was run for 1 minute at 48°C, 60 minutes at 42°C and 5 minutes at 95°C. Subsequently, the cDNA produced was amplified by PCR reaction. Next, 4 μL of 5X PCR buffer, 4 μL of magnesium chloride, 2 μL of customized forward primers mixture (Table 1), 0.7 μL of *Taq* polymerase and 9.3 μL of cDNA were mixed and run at specified time and temperature.

**Table 1:** List of genes with the primer and product size for GeXP multiplex analysis

**Gene**	**Accession number**	**Product size**	**Forward primer sequence**
*Beta actin*	NM_01101	230	GATCATTGCTCCTCCTGAGC
*SOD1*	NM_000454	320	TGGGGACAATACACAAGG
*SOD2*	NM_000636	330	AAAGGAGAGTTGCTGGAG
*Akt1*	NM_001014431	197	GAGGAGATGGACTTCCGGTC
*NF*-*κB*	NM_001077493	204	GCGGGCGTCTAAAATTCTG
*p53*	NM_001126117	168	GGGGAGCAGGGCTCA
*p38 MAPK*	NM_001315	247	TTCAGTCTTTGACTCAGATGCC
*Catalase*	NM_001752	350	GGCAGCTATGTGAGAGCC

Forward universal primer sequence (AGGTGACACTATAGAATA).

### GeXP multiplex analysis

The GenomeLab GeXP genetic analysis system (Beckman Coulter, USA) was used to examine the expression level of genes involved in apoptosis pathway. The forward and reverse primers were supplied by First Base Ltd. (Selangor, Malaysia). The genes and their primer sequences were listed in Table 1. Briefly, 1 μL of PCR product was mixed with 38.5 μL of sample loading solution and 0.5 μL of DNA size standard, and added into sample plate to start the sample run using GeXP Genetic Analysis System (Beckman Coulter, USA). The amplified fragments were separated according to their respective size by capillary gel electrophoresis in the GeXP system. Results were analyzed using the Fragment Analysis module of the GeXP system software and eXpress Profiller software. The normalization was performed using beta actin.

### Statistical analysis

Data were represented as mean ± SD of at least three independent experiments. Data were analyzed using IBM SPSS version 20. Statistical test one way ANOVA and Tukey post hoc test were conducted for pairwise comparisons. *P* value less than 0.05 was considered statistically significant.

## Results

### Cytotoxic properties of EADs on MCF-7 cells

**Figure 1:**
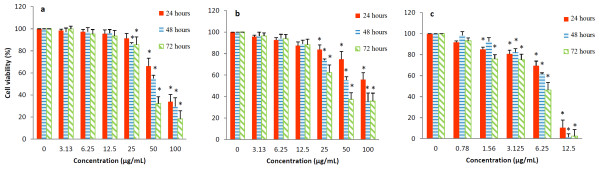
**Effect of EADs on the viability of MCF-7 cells as determined by MTT assay.** Cytotoxic effect of EADs was investigated on **(a)** breast adenocarcinoma cell line, MCF-7 and **(b)** non-neoplastic mouse fibroblast cell line, 3 T3. The result indicated that EADs was relatively non-selective towards cancerous cell line. Antiproliferative effect of EADs was compared to reference drug, tamoxifen on MCF-7 cell line **(Figure 1c)**. Data are represented as mean percentage of viable cells in bars ± SD of at least three replicates in three independent tests. An asterisk * indicates statistically significant different from untreated control (*P* < 0.05).

As shown in Figure 1, significant cytotoxic effect of EADs in MCF-7 cells was noted at 25, 50 and 100 μg/mL compared to the control at various time points (*P* < 0.05). The cytotoxic effect was time-and dose-dependent. Treatment with EADs at 25 and 50 μg/mL reduced the cell viability from 86.3% to 29.6% and 85.5% to 18.3%, respectively, from 48 to 72 hours (*P* < 0.05). IC_50_ values of EADs were 76 ± 2.3, 58 ± 0.7 and 39 ± 3.6 μg/mL, respectively, at 24, 48 and 72 hours. Based on the cytotoxic effect, 25 and 50 μg/mL of EADs, and incubation time of 24 and 48 hours were selected for further analysis.

### Morphological changes of MCF-7 cells following treatment with EADs

**Figure 2:**
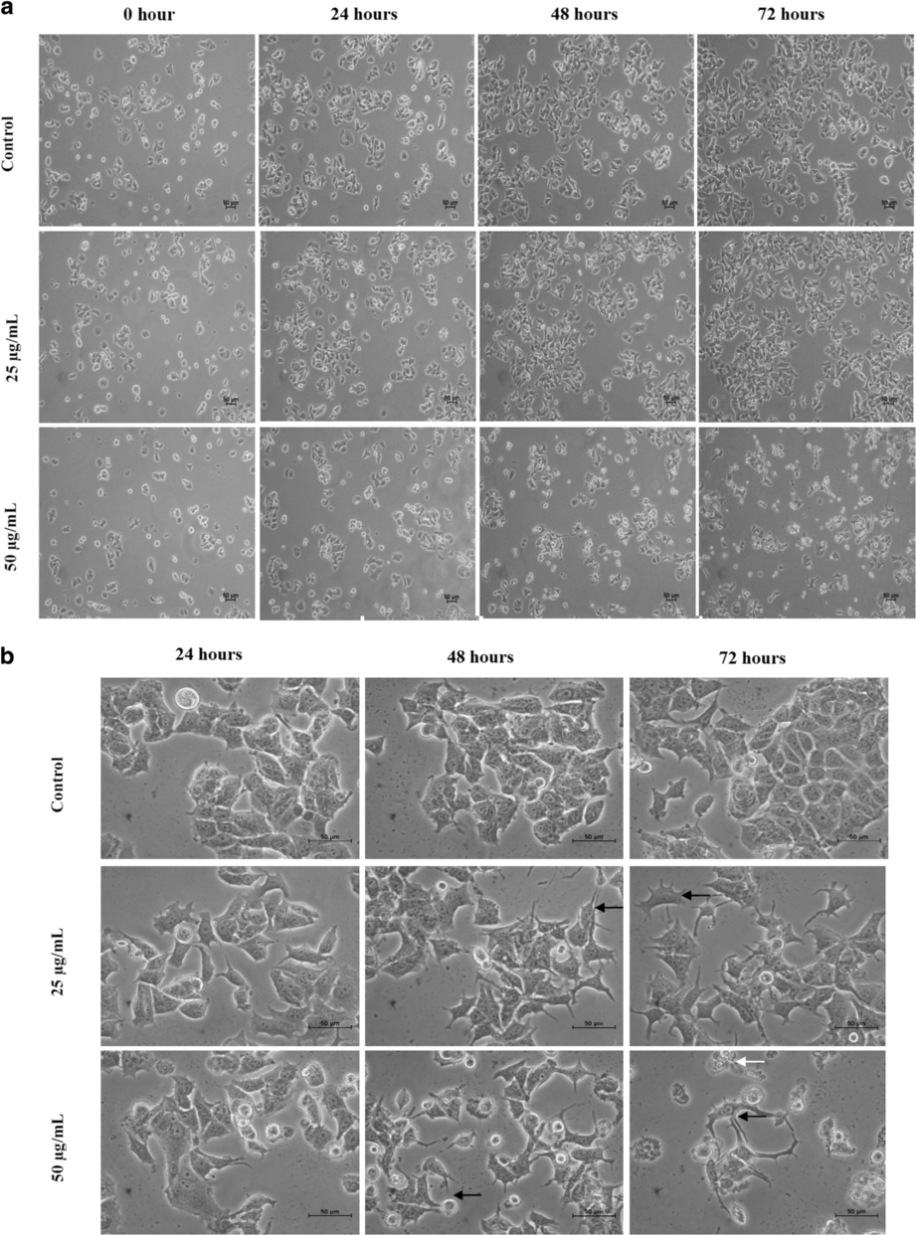
**Morphological changes of MCF-7 cells treated with EADs observed under an inverted light microscope.** The cells exhibited morphological changes and characteristics of apoptosis such as cell shrinkage and rounding (black arrow), and detachment from the substatum (white arrow). Decrease in cell population was noted with the increase in the concentration of the extract. **(a)** 100X magnification **(b)** 400X magnification

The cell number reduced at 50 μg/mL of EADs at 72 hours. Cell detachment, cell rounding, cytoplasmic condensation and cell shrinkage were observed at 48 and 72 hours in MCF-7 cells treated with 50 μg/mL of EADs (Figure 2). At 25 μg/mL of EADs, cell shrinkage and cytoplasmic condensation were noted but the cell number increased over time.

### EADs induced cell cycle arrest in MCF-7 cells

**Figure 3:**
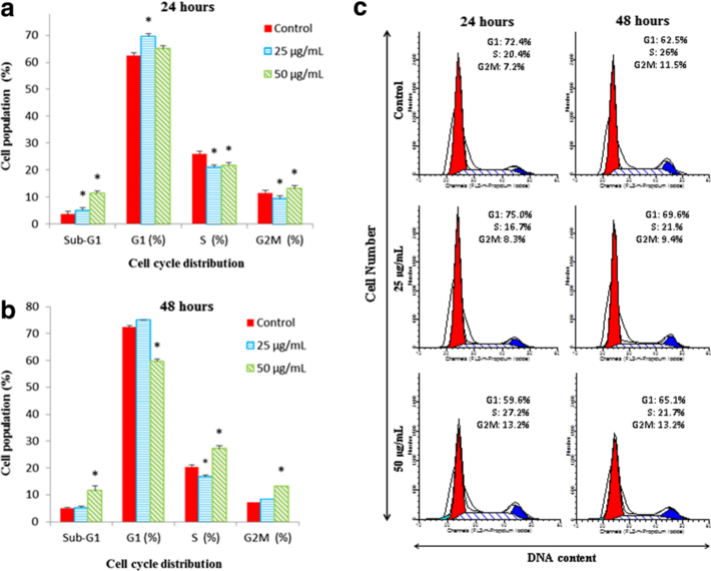
**Cell cycle analysis of MCF-7 breast cancer cells treated with EADs at 24 and 48 hours.** Effects of EADs on the cell cycle distribution in MCF-7 cells were analysed using flowcytometry analysis. Bar charts representing the percentage of cell populations in MCF-7 cells treated with EADS for **(a)** 24 hours and **(b)** 48 hours. DNA histogram **(Figure 3c)** displayed cell cycle phase distribution of control and EADs-treated cells at 24 and 48 hours. The data are presented as mean ± standard deviation of three replicates in three independent tests. An asterisk * indicates statistically significant different from untreated control (*P* < 0.05).

The cell cycle phase distribution of MCF-7 cells treated with EADs at 24 and 48 hours is depicted in Figure 3. The cell cycle arrest by EADs was time- and concentration-dependent. At 24 and 48 hours, an increase in cell population in G_1_ at 25 μg/mL of EADs was noted (*P* < 0.05). On the other hand, 50 μg/mL of EADs at 24 hours elevated the number of cells in S and G_2_/M compared to control, accompanied by a decline in G_1_ phase cell population (*P* < 0.05). Meanwhile, at 48 hours, G_2_/M phase cell population was ascended compared to the control following treatment with 50 μg/mL of EADs (*P* < 0.05). Increase in the population of cells at sub-G_1_ phase was observed following treatment with EADs (*P* < 0.05).

### EADs induced apoptosis in MCF-7 cells

**Figure 4:**
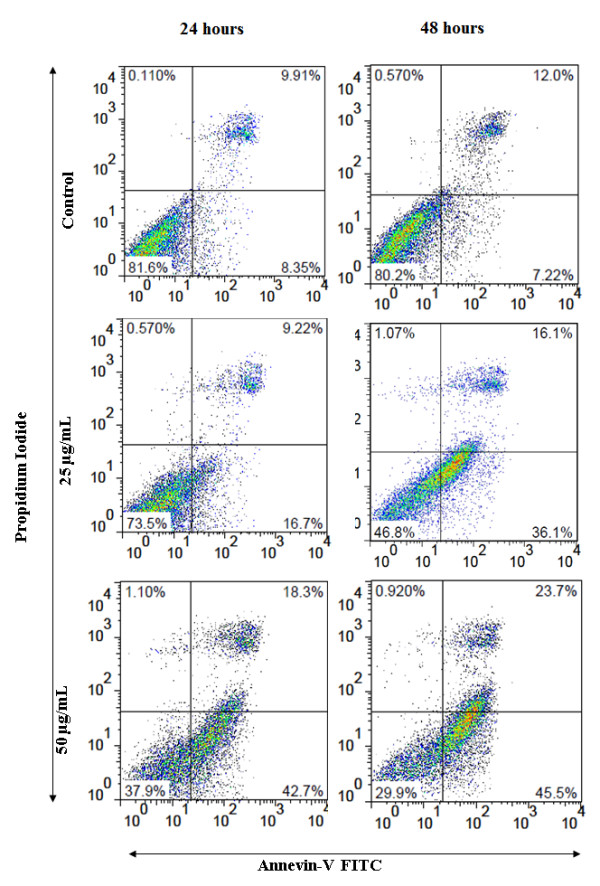
**Induction of apoptosis in MCF-7 cells by EADs determined using Annexin V-PI flowcytometry technique.** The data representing three independent tests which displayed similar results. The lower left quadrant represents intact viable cells (Annexin-FITC and PI negative). The lower right quadrant represents early apoptotic cells (Annexin-FITC positive and PI negative). The upper right region represents late apoptotic cells or secondary necrotic cells (Annexin-FITC and PI positive). The data are presented as dot plots of Annevin V/FITC against PI of at least three independent tests.

Induction of apoptosis by EADs was quantitatively determined by Annexin V-FITC and propidium iodide fluorescence staining. The percentage of early apoptotic cells increased in a dose and time dependent manner (Figure 4). At 24 hours, the early apoptotic cells increased from 16.7% at 25 μg/mL to 42.7% at 50 μg/mL of EADs compared to 8.3% in the control. The number of late apoptotic cells increased from 9.9% to 18.3% in 50 μg/ml of EADS (*P* < 0.05). At 48 hours, the number of early apoptotic cells increased to 36.1% at 25 μg/mL of EADs, and elevated to 45.5% at 50 μg/mL of EADs.

### EADs altered the expression of oxidative stress pathway related genes

**Figure 5:**
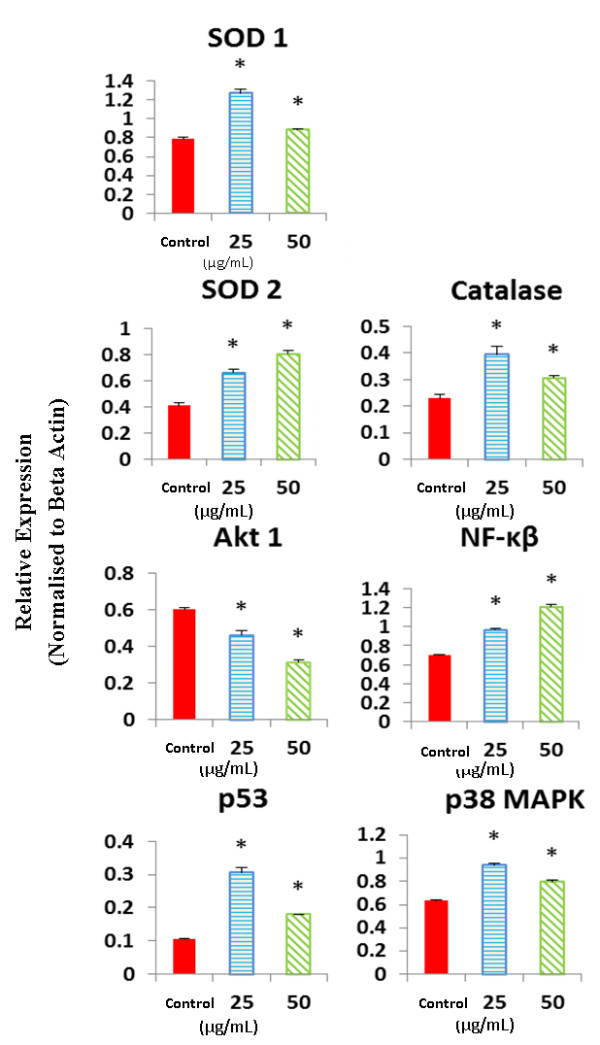
**Expression level of genes involved in oxidative stress-induced apoptotic pathway following treatment with EADs using GeXP analysis system.** The data are presented as mean ± standard deviation of three replicates in at least three independent tests. An asterisk * indicates statistically significant different from untreated control (*P* < 0.05).

The MCF-7 cells treated with 25 and 50 μg/mL of EADs significantly upregulated the expression level of *SOD1*, *SOD2*, *p53*, *p38 MAPK*, *catalase* and *NF*-*κB* genes but downregulated *Akt1* (*P* < 0.05) compared to the control (Figure 5).

## Discussion

Cancer cells evolve to avoid apoptosis-inducing signaling pathway in order to survive [40]. Thus, induction of apoptosis in cancer cells can be a promising treatment method in cancer therapy. Natural-derived products, regardless of crude extracts or isolated active compounds, had drawn growing attention as agent in cancer therapy, due to their ability to modulate apoptosis [41-43].

In this study, EADs has been shown to be cytotoxic and inhibit the proliferation of MCF-7 cells in a time- and dose-dependent manner. The cytotoxic property may be due to the presence of phytochemicals such as saponins, triterpenes, tannins and polyphenolic compounds in the extract [37]. Man *et al*. [44] reported that saponins exhibited anti-tumorigenic effects via multiple anticancer pathways because of the great diversity of their structures. For instance, a steroidal saponin known as Dioscin was described to inhibit tumor through induction of oxidative stress [45]. In addition, triterpenes from *Antrodia camphorata* were found to exhibit cytotoxic effect towards HT-29 human colon cancer cells [46]. Polyphenolic compounds exist in plants are associated with anticancer activity by interaction with key enzymes in cellular signaling pathways, cell cycle, apoptosis and metastasis [47,48].

The treatment of EADs elicited non-phase specific cell cycle arrest in MCF-7 cells. For instance, at 25 μg/mL of EADs, cell cycle arrest at G_1_ at 48 hours was noted. On the other hand, at 50 μg/mL of EADs, the cell cycle arrest was at S and G_2_M at 24 hours, but G_2_M cell cycle arrest at 48 hours. The cell cycle phase non-specific nature of EADs denotes that it kills tumor cells in either resting or dividing state. The non-specific phase drugs are among the most effective drugs against slow-growing tumors [49]. Ozawa *et al*. [50] demonstrated that the action of a non-phase specific antitumor agent is basically dependent on the concentration and time. This finding is in accordance with the cytotoxicity of EADs.

MCF-7 cells treated with EADs exhibited certain apoptotic features such as cell rounding, cell shrinkage and cytoplasmic condensation, and also the presence of sub-G1 phase population. The induction of apoptosis was then further confirmed by flowcytometric Annexin V-FITC/PI. One of the hallmarks of apoptosis is the externalization of phospholipid phosphatidylserine (PS) by translocation from the inner to outer layer of plasma membrane for recognition of phagocytes during early stage of apoptosis [51]. Hence, phosphatidylserine can serve as specific target for the detection of early apoptotic cells. Annexin V-FITC which has high binding affinity for phosphatidylserine is appropriate conjugate for identification of early stage apoptosis [52,53]. Simultaneously, propidium iodide is included for dye exclusion to differentiate between apoptotic and necrotic cells [54].

Interestingly, other prominent characteristics of apoptosis such as membrane blebbing, DNA fragmentation and formation of apoptotic bodies were absent. MCF-7 cells are previously reported lack in caspase-3, an important component in the cascade of apoptosis, due to deletion of a 47 base pair in the exon 3 of the caspase gene [55]. The caspase-3 deficient MCF-7 cells do not display some typical morphological characteristics of apoptosis such as chromatin condensation, DNA fragmentation and membrane blebbing [56]. During normal condition in apoptosis, caspase-3 is activated and responsible for morphological and biochemical changes related to the apoptosis execution [57]. It is speculated that other caspases such as caspase-6 or caspase-7 or caspase-independent pathway are involved in apoptosis induced by EADs [58].

Many anticancer agents induced apoptotic cell death by introducing oxidative stress to a threshold that compromises cell viability, disturbing the equilibrium between ROS and antioxidants within cancer cells [59]. From the GeXP analysis data, the expression of *SOD1*, *SOD2*, and *catalase* genes was upregulated. It is postulated that the antioxidant defense system in MCF-7 cells is triggered in response to increase cellular oxidative stress generated by EADs. Superoxide dismutases (SODs) act as the sole enzyme that dismutates superoxide radicals (O_2_^-^) into hydrogen peroxide (H_2_O_2_) and oxygen (O_2_). SOD1 is mainly located in the cytosol and intermembrane space of mitochondria while SOD2 is present in the matrix of mitochondria [16]. The product of SODs action, H_2_O_2_, is metabolized by an antioxidant known as catalase into water and oxygen [60]. In this case, *SOD1*, *SOD2* and *catalase* were upregulated in order to scavenge the elevating level of ROS induced by EADs. Nonetheless, in spite of the protective mechanism of the antioxidants, MCF-7 cells still underwent apoptosis. Hence, it is believed that the ROS level induced by EADs was high and has surpassed the antioxidant capacity, leading to apoptosis in MCF-7 cells [59,61].

In the present study, *Akt1* expression was downregulated in a concentration-dependent manner suggesting the involvement of Akt pathway in EADs-induced apoptosis. Previous studies have shown that apoptosis in MCF-7 cells was related to the inhibition of Akt signaling pathway after treatment with Wogonin or retinoic-acid [62,63]. Generally, Akt is a serine-threonine kinase that facilitates the control of balance between survival and apoptosis. Oxidative stress has been associated with the regulation of Akt pathway [64,65]. Studies have reported that in response to oxidative stress, Akt can be downregulated and it is important in apoptosis process [66]. Akt signalling pathway deregulation in cancer cells has been one of the targets in the search of potential cancer treatment [67].

In this study, upregulation of *p53* level was noted, which is possibly related to oxidative stress. Other than antioxidant defense, cells counterbalance the effect of oxidative stress by activation of p53-dependent pathways. p53 is a nuclear transcription factor that can be activated in response to oxidative stress to promote apoptosis by regulation of multitude of downstream effectors. Once it is activated, cell cycle is arrested for DNA repair process to restore normal cell function [68]. We propose that EADs-induced oxidative stress will increase the p53 level and subsequently lead to the non-phase specific cell cycle arrest in MCF-7 cells. However, if cells are not able to overcome the oxidative stress damage and DNA damage cannot be repaired, p53 becomes a mediator to induce apoptosis [68,69].

EADs treatment in MCF-7 cells was also found to upregulate *NF*-*κB* expression. It is believed that increased activity of NF-κB in EADs-induced apoptosis is again due to oxidative stress. The nuclear transcription factor NF-κB regulates genes involved in a number of biological processes such as inflammation, cell survival, cell differentiation and cell growth [70]. NF-κB enhances the pro-inflammatory and anti-apoptotic genes expression, and acts as a protective barrier for cells against oxidative stress. However, NF-κB has also been associated with apoptosis and brings to activation of certain apoptosis-related genes [58]. Furthermore, many studies supported the pro-apoptotic effect exerted by NF-κB in response to oxidative stress [71]. It has been concluded that pro-apoptotic or anti-apoptotic effect of NF-κB depends on stimuli received, signaling pathway interactions, transcriptional regulation and function of genes it modulates [72]. ROS activate NF-κB via the dissociation of IκB from NF-κB through phosphorylation, thereby enable NF-κB to enter nucleus and activate transcription by binding to DNA [73].

Another intracellular signaling molecule that is involved in regulation of oxidative stress is p38 MAPK which belongs to MAPK superfamily. In accordance, a marked increase in gene expression of *p38 MAPK* in MCF-7 cells treated with EADs was observed. The p38 MAPK strongly responds to stress-inducing signals such as oxidative stress and cause apoptosis as a result of cellular injuries [16]. The p38 MAPK plays a role in regulation of cellular biological functions like inflammation, proliferation, differentiation, survival. [74,75]. This pathway has been known as tumor suppressor because it is often activated by cellular stress and control signals that inhibit proliferation or enhance apoptosis [76]. Investigation of ROS activated p38 MAPK has been carried out widely. In addition, p38 MAPK and Akt pathways were found to be interconnected in numerous cases. For example, a study reported that suppression of Akt signaling pathway excites p38 MAPK related apoptosis and vice versa [77]. Our result also indicated that oxidative stress stimulates downregulation of Akt and facilitates upregulation of p38 MAPK, hence give rise to apoptosis in EADs treated MCF-7 breast cancer cells.

Nonetheless, several studies documented that Akt pathway is involved in the activation of NF-κB pathway under the treatment of TNF-α and growth factor [78]. However, it has also been demonstrated that NF-κB pathway may function independently from Akt pathway. NF-κB binding and transcription activity can still be activated despite of the inhibition of the Akt pathway [79]. Hence, it is postulated that although some crosstalks exist between Akt pathway and NF-κB pathway in response to EADs-induced oxidative stress in MCF-7 cells, but it appears that these two pathways can act independently. As discussed above, in response to oxidative stress, p53 is able to exert pro-apoptotic effect. Furthermore, it has been shown that p38 MAPK can phosphorylate p53, and play part in regulation of p53 expression under stress situation by stabilizing the p53 protein.

## Conclusions

In summary, EADs was found to exhibit cytotoxicity towards MCF-7 cell line possibly via introduction of oxidative stress that activates Akt, NF-κB, p53, and p38 MAPK signaling pathway. It shows the potential of EADs to be developed into an anticancer agent. Nevertheless, the shift of attention towards bioactive compound that are responsible for the anti-breast cancer activity of EADs, and understanding of its mechanism of action are utmost essential to discover the potential of the extract in breast cancer intervention.

## Abbreviations

MTT: 3-(4,5-dimethylthiazol-2-yl)-2,5 diphenyltetrazolium bromide; ANOVA: Analysis of variance; DNA: Deoxyribonucleic acid; DMSO: Dimethyl sulfoxide; ELISA: Enzyme-linked immunosorbent assay; EADs: Ethyl acetate extract of *D. suffruticosa*; p38 MAPK: P38 mitogen-activated protein kinases; NF-κB: Nuclear factor-kappaB; TNF-α: Tumor necrosis factor alpha; PBS: Phosphate buffered saline; Akt: Protein 53 (p53).protein kinase B; ROS: Reactive oxygen species.

## Competing interests

The authors declare that they have no competing interests.

## Authors’ contributions

YST carried out the study and prepared the manuscript. YST and JBF collected and interpreted the data. NA and JBF contributed to the preparation of plant extract. MUI and NI contributed to GeXP analysis. LSY, RA, YKC and MI contributed to the design and conception of the study and interpretation of data. LSY critically revised manuscript and codirected with MI who supervised and provided reagents and facilities. All authors have read and approved the manuscript for publication.

## Pre-publication history

The pre-publication history for this paper can be accessed here:

http://www.biomedcentral.com/1472-6882/14/55/prepub

## References

[B1] United States Cancer Statistics 1999–2007 Incidence and Mortality Web-based Reporthttp://www.cdc.gov/uscs

[B2] ACS Cancer Facts and Figures 2012 2012 Cancers with Increasing Incidence Trends http://www.cancer.org/acs/groups/content/@epidemiologysurveilance/documents/document/acspc-031941.pdf

[B3] Siegel R Naishadham D Jemal A Cancer statistics CA Cancer J Clin 2012 62 1 10-29 http://dx.doi.org/10.3322/caac.20138 10.3322/caac.20138 22237781

[B4] Forman D Ward E Ferlay M Jemal A Bray F Global cancer statistics CA Cancer J Clin 2011 61 2 69 90 http://dx.doi.org/10.3322/caac.20107 10.3322/caac.20107 21296855

[B5] Kaufmann SH Gores GJ Apoptosis in cancer: cause and cure BioEssays 2000 22 11 1007 1017 http://dx.doi.org/10.1002/1521-1878(200011)22:11<1007::AID-BIES7>3.0.CO;2-4 10.1002/1521-1878(200011)22:11<1007::AID-BIES7>3.0.CO;2-4 11056477

[B6] McGill G Fisher DE Apoptosis in tumorigenesis and cancer therapy Frontiers in Biosci: a J and virtual Lib 1997 2 d353 d379 http://europepmc.org/abstract/MED/9230063 10.2741/a197 9230063

[B7] Sledge GW Jr Miller KD Exploiting the hallmarks of cancer: the future conquest of breast cancer Eur J Cancer 2003 39 12 1668 1675 http://dx.doi.org/10.1016/S0959-8049(03)00273-9 10.1016/S0959-8049(03)00273-9 12888360

[B8] Kasibhatla S Tseng B Why target apoptosis in cancer treatment? Mol Cancer Ther 2003 2 6 573 580 http://mct.aacrjournals.org/content/2/6/573.abstract 12813137

[B9] Wong RSY Apoptosis in cancer: from pathogenesis to treatment J Exp Clin Cancer Res 2011 30 87 http://www.jeccr.com/content/30/1/87 10.1186/1756-9966-30-87 21943236 PMC3197541

[B10] Kroemer G Dallaporta B Resche-Rigon M The mitochondrial death/life regulator in apoptosis and necrosis Annu Rev Physiol 1998 60 619 642 http://dx.doi.org/10.1146/annurev.physiol.60.1.619 10.1146/annurev.physiol.60.1.619 9558479

[B11] Lowe SW Lin AW Apoptosis in cancer Carcinogenesis 2000 21 3 485 495 http://dx.doi.org/10.1093/carcin/21.3.485 10.1093/carcin/21.3.485 10688869

[B12] Simic MG Bergtold DS Karam LR Generation of oxy radicals in biosystems Mutation Res/Fundamental and Mol Mech of Mutagenesis 1989 214 1 3 12 http://dx.doi.org/10.1016/0027-5107(89)90192-9 10.1016/0027-5107(89)90192-9 2671698

[B13] Kryston TB Georgiev AB Pissis P Georgakilas AG Role of oxidative stress and DNA damage in human carcinogenesis Mutation Res/Fundamental and Mol Mech of Mutagenesis 2011 711 1–2 193 201 http://dx.doi.org/10.1016/j.mrfmmm.2010.12.016 10.1016/j.mrfmmm.2010.12.016 21216256

[B14] Sosa V Moliné T Somoza R Paciucci R Kondoh H Lleonart ME Oxidative stress and cancer: an overview Ageing Res Rev 2013 12 1 376 390 http://dx.doi.org/10.1016/j.arr.2012.10.004 10.1016/j.arr.2012.10.004 23123177

[B15] Dreher D Junod AF Role of oxygen free radicals in cancer development Eur J Cancer 1996 32 1 30 38 http://dx.doi.org/10.1016/0959-8049(95)00531-5 10.1016/0959-8049(95)00531-5 8695238

[B16] Valko M Rhodes CJ Moncol J Izakovic M Mazur M Free radicals, metals and antioxidants in oxidative stress-induced cancer Chem Biol Interact 2006 160 1 1 40 http://dx.doi.org/10.1016/j.cbi.2005.12.009 10.1016/j.cbi.2005.12.009 16430879

[B17] Wen J You K-R Lee S-Y Song C-H Kim D-G Oxidative stress-mediated apoptosis: the anticancer effect of the sesquiterpene lactone parthenolide J Biol Chem 2002 277 41 38954 38964 http://dx.doi.org/10.1074/jbc.M203842200 10.1074/jbc.M203842200 12151389

[B18] Afzal S Jensen S Sørensen J Henriksen T Weimann A Poulsen H Oxidative damage to guanine nucleosides following combination chemotherapy with 5-fluorouracil and oxaliplatin Cancer Chemother Pharmacol 2012 69 2 301 307 http://dx.doi.org/10.1007/s00280-011-1700-2 10.1007/s00280-011-1700-2 21710244

[B19] Alexandre J Hu Y Lu W Pelicano H Huang P Novel action of paclitaxel against cancer cells: bystander effect mediated by reactive oxygen species Cancer Res 2007 67 8 3512 3517 http://dx.doi.org/10.1158/0008-5472.can-06-3914 10.1158/0008-5472.CAN-06-3914 17440056

[B20] Nazarewicz RR Zenebe WJ Parihar A Larson SK Alidema E Choi J Ghafourifar P Tamoxifen induces oxidative stress and mitochondrial apoptosis via stimulating mitochondrial nitric oxide synthase Cancer Res 2007 67 3 1282 1290 http://dx.doi.org/10.1158/0008-5472.can-06-3099 10.1158/0008-5472.CAN-06-3099 17283165

[B21] Graham JG Quinn ML Fabricant DS Farnsworth NR Plants used against cancer – an extension of the work of Jonathan Hartwell J Ethnopharmacol 2000 73 347 377 http://www.sciencedirect.com/science/article/pii/S037887410000341X 10.1016/S0378-8741(00)00341-X 11090989

[B22] Farnsworth NR The Role of Etnopharmacology in Drug Development 1990 England: John Wiley Chichester http://www.ncbi.nlm.nih.gov/pubmed/2086037

[B23] Liu JH Activation of apoptosis pathways by different classes of anticancer drugs 2001 Hubei: University of Ulm http://vts.uni-ulm.de/docs/2001/798/vts_798.pdf

[B24] Hannun YA Apoptosis and the dilemma of cancer chemotherapy Blood 1997 89 6 1845 1853 http://bloodjournal.hematologylibrary.org/content/89/6/1845.short 9058703

[B25] Debatin K-M Activation of apoptosis pathways by anticancer treatment Toxicol Lett 2000 112–113 0 41 48 http://dx.doi.org/10.1016/S0378-4274(99)00252-0 10.1016/s0378-4274(99)00252-0 10720711

[B26] Hickman J Apoptosis induced by anticancer drugs Cancer Metastasis Rev 1992 11 2 121 139 http://dx.doi.org/10.1007/BF00048059 10.1007/BF00048059 1327566

[B27] Yeh CC Tseng CN Yang JI Huang HW Fang Y Tang JY Chang FR Chang HW Antiproliferation and induction of apoptosis in Ca9-22 oral cancer cells by ethanolic extract of gracilaria tenuistipitata Molecules 2012 17 9 10916 10927 http://www.mdpi.com/1420-3049/17/9/10916 22968475 10.3390/molecules170910916 PMC6269058

[B28] Yoo C-B Han K-T Cho K-S Ha J Park H-J Nam J-H Kil U-H Lee K-T Eugenol isolated from the essential oil of Eugenia caryophyllata induces a reactive oxygen species-mediated apoptosis in HL-60 human promyelocytic leukemia cells Cancer Lett 2005 225 1 41 52 http://dx.doi.org/10.1016/j.canlet.2004.11.018 10.1016/j.canlet.2004.11.018 15922856

[B29] Altinok B Sunguroglu A Karadag A Gurleyik E Ozkan T Aydos OS Avci A Antiproliferative, apoptotic and antioxidant activities of wheatgrass (Triticum aestivum L.) extract on CML (K562) cell line Turkish J of Med Sci 2011 41 4 657 663 http://journals.tubitak.gov.tr/medical/issues/sag-11-41-4/sag-41-4-13-0912-425.pdf

[B30] Xiao D Zeng Y Prakash L Badmaev V Majeed M Singh SV Reactive oxygen species-dependent apoptosis by gugulipid extract of ayurvedic medicine plant commiphora mukul in human prostate cancer cells is regulated by c-Jun N-terminal kinase The Am Soc for Pharmacol and Exp Ther 2010 79 499 507 http://dx.doi.org/10.1124/mol.110.068551 10.1124/mol.110.068551 PMC3061364 21115635

[B31] Chong KY Tan HTW Corlett RT A checklist of the total vascular plant flora of Singapore: native, naturalised and cultivated species. Raffles Museum of Biodiversity Research, National University of Singapore 2009 Singapore: Raffles Museum of Biodiversity Research, National University of Singapore 273 http://rmbr.nus.edu.sg/raffles_museum_pub/flora_of_singapore_tc.pdf

[B32] U.S. Department Agriculture ARS National Genetic Resources Program 2011 USA: Germplasm Resources Information Network (GRIN). Online searchable database http://www.ars-grin.gov/cgi-bin/npgs/html/index.pl

[B33] Ahmad FB Holdsworth DK Traditional medicinal plants of Sabah, Malaysia part III. The Rungus people of Kudat Int J Pharmacogn 1995 33 262 264 http://dx.doi.org/10.3109/13880209509065377 10.3109/13880209509065377

[B34] Hanum F Hamzah N The use of medicinal plant species by the Temuan tribe of Ayer Hitam Forest, Selangor, Peninsular Malaysia Pertanika J of Tropical Agri 1999 22 2 85 94 http://psasir.upm.edu.my/3802/1/The_Use_of_Medicinal_Plant_Species_by_the_Temuan_Tribe_of_Ayer_Hitam.pdf

[B35] Wiart C Mogana S Khalifah S Mahan M Ismail S Buckle M Narayana AK Sulaiman M Antimicrobial screening of plants used for traditional medicine in the state of Perak, Peninsular Malaysia Fitoterapia 2004 75 1 68 73 http://www.sciencedirect.com/science/article/pii/S0367326X03002089 10.1016/j.fitote.2003.07.013 14693223

[B36] Muliawan SY Effect of Dillenia suffruticosa extract on dengue virus type 2 replication Universa Med 2008 27 1 1 5 http://www.univmed.org/wp-content/uploads/2011/02/dr.sylvia.pdf

[B37] Armania N Yazan L Musa SN Ismail IS Foo JB Chan KW Noreen H Hisyam AH Zulfahmi S Ismail M Dillenia suffruticosa exhibited antioxidant and cytotoxic activity through induction of apoptosis and G2/M cell cycle arrest J Ethnopharmacol 2013 146 2 525 535 http://dx.doi.org/10.1016/j.jep.2013.01.017 10.1016/j.jep.2013.01.017 23353897

[B38] Wang CZ Li XL Wang QF Mehendale SR Yuan CS Selective fraction of Scutellaria baicalensis and its chemopreventive effects on MCF-7 human breast cancer cells Phytomedicine 2010 17 1 63 68 http://dx.doi.org/10.1016/j.phymed.2009.07.003 10.1016/j.phymed.2009.07.003 19836937 PMC2789205

[B39] Mosmann T Rapid colorimetric assay for cellular growth and survival: application to proliferation and cytotoxicity assays J Immunol Methods 1983 65 1–2 55 63 http://dx.doi.org/10.1016/0022-1759(83)90303-4 6606682 10.1016/0022-1759(83)90303-4

[B40] Hanahan D Weinberg Robert A Hallmarks of cancer: the next generation Cell 2011 144 5 646 674 http://dx.doi.org/10.1016/j.cell.2011.02.013 10.1016/j.cell.2011.02.013 21376230

[B41] Fulda S Modulation of apoptosis by natural products for cancer therapy Planta Med 2010 76 EFirst 1075 1079 http://dx.doi.org/10.1055/s-0030-1249961 20486070 10.1055/s-0030-1249961

[B42] Bailly C Ready for a comeback of natural products in oncology Biochem Pharmacol 2009 77 9 1447 1457 http://dx.doi.org/10.1016/j.bcp.2008.12.013 10.1016/j.bcp.2008.12.013 19161987

[B43] Taraphdar AK Roy M Bhattacharya RK Natural products as inducers of apoptosis: implication for cancer therapy and prevention Curr Sci 2001 80 11 1387 1396 http://www.iisc.ernet.in/currsci/jun102001/1387.pdf

[B44] Man S Gao W Zhang Y Huang L Liu C Chemical study and medical application of saponins as anti-cancer agents Fitoterapia 2010 81 7 703 714 http://dx.doi.org/10.1016/j.fitote.2010.06.004 10.1016/j.fitote.2010.06.004 20550961

[B45] Zhiyu W Yue C Neng W Mei WD Wei LY Feng H Gang SJ De Po Y Yuan GX Jian-Ping C Dioscin induces cancer cell apoptosis through elevated oxidative stress mediated by downregulation of peroxiredoxins Cancer Biol & Therapy 2012 13 3 138 147 http://www.landesbioscience.com/journals/cbt/article/18693/10.4161/cbt.13.3.18693 22231406

[B46] Yeh CT Rao YK Yao CJ Yeh CF Li CH Chuang SE Luong JHT Lai GM Tzeng YM Cytotoxic triterpenes from Antrodia camphorata and their mode of action in HT-29 human colon cancer cells Cancer Lett 2009 285 1 73 79 http://dx.doi.org/10.1016/j.canlet.2009.05.002 10.1016/j.canlet.2009.05.002 19477064

[B47] Lamoral-Theys D Pottier L Dufrasne F Neve J Dubois J Kornienko A Kiss R Ingrassia L Lamoral-Theys D Pottier L Dufrasne F Neve J Dubois J Kornienko A Kiss R Ingrassia L Natural polyphenols that display anticancer properties through inhibition of kinase activity Curr Med Chem 2010 17 814 812 825 http://dx.doi.org/10.2174/092986710790712183 20156174 10.2174/092986710790712183

[B48] Kuttan G Kumar KB Guruvayoorappan C Kuttan R Antitumor, anti-invasion, and antimetastatic effects of curcumin Adv Exp Med Biol 2007 595 173 184 http://link.springer.com/chapter/10.1007%2F978-0-387-46401-5_6 10.1007/978-0-387-46401-5_6 17569210

[B49] Barton-Burke M Wilkes GM Cancer Therapies 2006 10 United States of America: Jones and Barlett’s books

[B50] Ozawa S Sugiyama Y Mitsuhashi Y Kobayashi T Inaba M Cell killing action of cell cycle phase-non-specific antitumor agents is dependent on concentration-time product Cancer Chemother Pharmacol 1988 21 3 185 190 http://dx.doi.org/10.1007/bf00262767 3129204 10.1007/BF00262767

[B51] Fadok VA Voelker DR Campbell PA Cohen JJ DLB Henson PM Exposure of phosphatidylserine on the surface of apoptotic lymphocytes triggers specific recognition and removal by macrophages J Immunol 1992 148 2207 http://www.jimmunol.org/content/148/7/2207.long 1545126

[B52] Vermes I Haanen C Steffens-Nakken H Reutellingsperger C A novel assay for apoptosis flow cytometric detection of phosphatidylserine expression on early apoptotic cells using fluorescein labelled annexin V J Immunol Methods 1995 184 1 39 51 http://dx.doi.org/10.1016/0022-1759(95)00072-I 10.1016/0022-1759(95)00072-I 7622868

[B53] Zhang G Gurtu V Kain SR Yan G Early detection of apoptosis using a fluorescent conjugate of annexin V BioTechniques 1997 23 525 531 http://www.biotechniques.com/multimedia/archive/00011/97233pf01_11715a.pdf 9298227 10.2144/97233pf01

[B54] Schutte B Nuydens R Geerts H Ramaekers F Annexin V binding assay as a tool to measure apoptosis in differentiated neuronal cells J Neurosci Methods 1998 86 1 63 69 http://dx.doi.org/10.1016/S0165-0270(98)00147-2 10.1016/S0165-0270(98)00147-2 9894786

[B55] Jänicke RU Sprengart ML Wati MR Porter AG Caspase-3 is required for DNA fragmentation and morphological changes associated with apoptosis J Biol Chem 1998 273 16 9357 9360 http://dx.doi.org/10.1074/jbc.273.16.9357 10.1074/jbc.273.16.9357 9545256

[B56] Jänicke R MCF-7 breast carcinoma cells do not express caspase-3 Breast Cancer Res Treat 2009 117 1 219 221 http://dx.doi.org/10.1007/s10549-008-0217-9 10.1007/s10549-008-0217-9 18853248

[B57] Mooney LM Al-Sakkaf KA Brown BL Dobson PR Apoptotic mechanisms in T47D and MCF-7 human breast cancer cells Br J Cancer 2002 87 8 909 917 http://europepmc.org/abstract/MED/12373608 10.1038/sj.bjc.6600541 12373608 PMC2376174

[B58] Kasibhatla S Genestier L Green DR Regulation of Fas-ligand expression during activation-induced cell death in T lymphocytes via nuclear factor κB J Biol Chem 1999 274 2 987 992 http://dx.doi.org/10.1074/jbc.274.2.987 10.1074/jbc.274.2.987 9873041

[B59] Kong Q Beel JA Lillehei KO A threshold concept for cancer therapy Med Hypotheses 2000 55 1 29 35 http://dx.doi.org/10.1054/mehy.1999.0982 10.1054/mehy.1999.0982 11021322

[B60] Kinnula VL Crapo JD Superoxide dismutases in malignant cells and human tumors Free Radic Biol Med 2004 36 6 718 744 http://dx.doi.org/10.1016/j.freeradbiomed.2003.12.010 10.1016/j.freeradbiomed.2003.12.010 14990352

[B61] Salganik RI The benefits and hazards of antioxidants: controlling apoptosis and other protective mechanisms in cancer patients and the human population J Am Coll Nutr 2001 20 5 464S 472S http://www.ncbi.nlm.nih.gov/pubmed/11603657 11603657 10.1080/07315724.2001.10719185

[B62] Miller WH Ratna S Rincón SVD Rousseau Retinoic acid-induced growth arrest of MCF-7 cells involves the selective regulation of the IRS-1/PI 3-kinase/AKT pathway Oncogene 2002 22 3353 3360 http://dx.doi.org/10.1038/sj.onc.1206485 10.1038/sj.onc.1206485 12776186

[B63] Diao Y Huang YQ Zhang GD Huang KF Wogonin induces apoptosis and down-regulates survivin in human breast cancer MCF-7 cells by modulating PI3K–AKT pathway Int Immunopharmacol 2012 12 2 334 341 http://dx.doi.org/10.1016/j.intimp.2011.12.004 10.1016/j.intimp.2011.12.004 22182776

[B64] Testa JR Bellacosa A AKT plays a central role in tumorigenesis Proc Natl Acad Sci 2001 98 20 10983 10985 http://dx.doi.org/10.1073/pnas.211430998 10.1073/pnas.211430998 11572954 PMC58668

[B65] Martindale JL Holbrook NJ Cellular response to oxidative stress: signaling for suicide and survival J of Cell Physiol 2002 192 1 15 http://dx.doi.org/10.1002/jcp.10119 10.1002/jcp.10119 12115731

[B66] Chang YJ Huang YP Li ZL Chen CH GRP78 Knockdown enhances apoptosis via the down-regulation of oxidative stress and Akt pathway after epirubicin treatment in colon cancer DLD-1 cells PLoS ONE 2012 7 4 e35123 http://dx.doi.org/10.1371/journal.pone.0035123s 10.1371/journal.pone.0035123 22529978 PMC3329422

[B67] Toker A Yoeli-Lerner M Akt signaling and cancer: surviving but not moving on Cancer Res 2006 66 8 3963 3966 http://dx.doi.org/10.1158/0008-5472.can-06-0743 10.1158/0008-5472.CAN-06-0743 16618711

[B68] Bates S Vousden KH Mechanisms of p53-mediated apoptosis Cell Mol Life Sci 1999 55 28 37 http://www.ncbi.nlm.nih.gov/pubmed/10065149sss 10.1007/s000180050267 10065149 PMC11147148

[B69] Robbins D Zhao Y Oxidative stress induced byMnSOD-p53 interaction: Pro- or anti-tumorigenic? J of Signal Transduct 2012 2012 1 13 http://dx.doi.org/10.1155/2012/101465 10.1155/2012/101465 PMC3189584 22007296

[B70] Sethi G Sung B Aggarwal BB Nuclear factor-κB activation: from bench to bedside Exp Biol and Med 2008 233 1 21 31 http://dx.doi.org/10.3181/0707-mr-196 10.3181/0707-MR-196 18156302

[B71] Aoki M Nata T Morishita R Matsushita H Nakagami H Yamamoto K Yamazaki K Nakabayashi M Ogihara T Kaneda Y Endothelial apoptosis induced by oxidative stress through activation of NF-κB: antiapoptotic effect of antioxidant agents on endothelial cells Hypertension 2001 38 1 48 55 http://dx.doi.org/10.1161/01.hyp.38.1.48 10.1161/01.HYP.38.1.48 11463759

[B72] Martin AG NFkB anti-apoptotic or pro-apoptotic, maybe both Cell Cycle 2010 9 16 3131 3132 http://www.mendeley.com/download/public/14520753/4840739732/054ce21125d09cd5018cfb8b4e375f6646499037/dl.pdf 10.4161/cc.9.16.12780 20724840

[B73] Janssen-Heininger YMW Poynter ME Baeuerle PA Recent advances torwards understanding redox mechanisms in the activation of nuclear factor κb Free Radic Biol Med 2000 28 9 1317 1327 http://dx.doi.org/10.1016/S0891-5849(00)00218-5 10.1016/S0891-5849(00)00218-5 10924851

[B74] Zarubin T Han J Activation and signaling of the p38 MAP kinase pathway Cell Res 2005 15 11 18 http://dx.doi.org/doi:10.1038/sj.cr.7290257 10.1038/sj.cr.7290257 15686620

[B75] Park K-R Nam D Yun H-M Lee S-G Jang H-J Sethi G Cho SK Ahn KS β-Caryophyllene oxide inhibits growth and induces apoptosis through the suppression of PI3K/AKT/mTOR/S6K1 pathways and ROS-mediated MAPKs activation Cancer Lett 2011 312 2 178 188 http://dx.doi.org/10.1016/j.canlet.2011.08.001 10.1016/j.canlet.2011.08.001 21924548

[B76] Massaoka MH Matsuo AL Figueiredo CR Farias CF Girola N Jacaranone induces apoptosis in melanoma cells via ROS-mediated downregulation of Akt and p38 MAPK activation and displays antitumor activity in vivo PLoS ONE 2012 7 6 e38698 http://dx.doi.org/10.1371/journal.pone.0038698 10.1371/journal.pone.0038698 22701695 PMC3368838

[B77] Gratton JP Morales-Ruiz M Kureishi Y Fulton D Walsh K Sessa WC Akt down-regulation of p38 signaling provides a novel mechanism of vascular endothelial growth factor-mediated cytoprotection in endothelial cells J Biol Chem 2001 276 32 30359 30365 http://dx.doi.org/10.1074/jbc.M009698200 10.1074/jbc.M009698200 11387313

[B78] Romashkova JA Makarov SS NF-κB is a target of AKT in anti-apoptotic PDGF signalling Nature 1999 401 86 90 http://dx.doi.org/10.1038/43474 10485711 10.1038/43474

[B79] Van Antwerp DJ Martin SJ Kafri T Green DR Verma IM Suppression of TNF-α-induced apoptosis by NF-κB Science 1996 274 5288 787 789 http://dx.doi.org/10.1126/science.274.5288.787 10.1126/science.274.5288.787 8864120

